# The Breakdown of the Hospital System

**Published:** 1889-03-23

**Authors:** P. J. Michelli

**Affiliations:** Secretary, Dreadnought Seamen's Hospital, Greenwich, S.E.


### THE BREAKDOWN OF THE HOSPITAL SYSTEM.

By Mr. P. J. MICHELLI, Secretary, Dreadnought Seamen's Hospital, Greenwich, S.E.

(*Continued from page* 358.)

THE title of Dr. Sutherland's paper is, as I have pointed out before, more alarming than accurate. It is not so much that the system has broken down as it is that there is in so many instances a want of system. A want of control over the administration of hospitals has landed the medical charities in their present difficulties. Nevertheless, Dr. Sutherland has made out a strong case for hospital reform.

That the medical assistance for the non-pauper poor is miserably inadequate and unevenly distributed has been proved beyond doubt; but that cannot be considered an excuse for reckless expenditure with the most remote possibility of meeting liabilities. There are many philanthropists who make efforts to supply additional bed accommodation, but there are few who give a thought to the curtailment of extravagance or the classification of those who are recipients of the benefits they provide. It is not too much to say that if the promoters of the Penny a week Fund raise £100,000 per annum from the working classes for the medical charities of London, the money will not so much go to defray the cost of maintaining the existing beds as it will be a direct incentive for many hospital managers to entail additional liabilities, to build new wings, etc., and so to leave the hospitals in the same financial position five years hence which they occupy to-day.

I have already raised my voice against erecting new small hospitals in the vicinity of the large general hospitals, but the extension of the existing great institutions before adequate means are forthcoming is just as much to be deprecated. It is in this perhaps as much as anything that we want a central bureau, some authority that will be able to exercise a healthy control upon the enthusiasm of those well meaning people who go into debt for the benefit of their fellow creatures, leaving their successors to settle the account, and upon those advertising members of the medical profession who are nowhere if not associated with and on the staff of a hospital, and for this purpose start one with the aid of their wealthier patients. The fact is that we all are dipping into the same pocket, but we are all spending the money we get out after our own lights, without any thought or control; indeed, pledging the credit of a generous public up to the hilt. To such an extent has this been recognised that a debt is looked upon as rather a valuable acquisition, and is boldly put forward as a virtuous plea. It is not that we have not got a handsome income, but simply that we are living beyond it.

The fear of injuring the voluntary system of medical relief is that which keeps many silent about the needy circumstances of our hospitals. These silent people see the charities going from bad to worse, and yet do nothing to better the existing state of affairs. Dr. Sutherland has plainly shown how many deserving patients are forced to go to the poor-law infirmary, and become paupers; and he has also shown how frequently those who are born and bred to the union, and feel no indignity at receiving poor-law relief, get into our voluntary charities; and, again, how many there are receiving free medical relief who could afford to pay, but who prefer to rob both the charity and the general practitioner. Surely reticence on such subjects as these is as uncharitable as it is weak. Is it not better that we should reform ourselves from within and court investigation than, that we should be reformed from without, which will be the inevitable consequence? This fact seems to be impressing itself on the minds of some who heretofore have not been the most anxious to show their lights before men.

It is strange indeed in these days to find anyone in favour of the letter or line system. Its only object is avowedly to induce those to subscribe who otherwise would not do so; but if this is the case, it also operates by helping to fill the beds of a hospital with unworthy objects, whose maintenance costs more than the subscription contributed by such narrow-minded and uncharitable persons, to the exclusion of really deserving cases. These supporters, who are so anxious for their *quid pro quo*, utilise their letters to gain admittance for their domestic servants and other persons for whom the charity is not intended.

When the whole question is reviewed from a common standpoint, much will be apparent that now it is impossible to unearth. Therefore let us have a properly constituted authority that shall have power to go thoroughly into the matter, and to suggest the best way of remedying the existing evil. The well-managed charities have nothing to hide, and a Royal Commission will break down the barriers which hinder a comprehensive view from being taken of the hospital question.

